# Barriers and Breakthroughs in Precision Oncology: A National Registry Study of *BRCA* Testing and PARP Inhibitor Uptake in Women from the National Gynae-Oncology Registry (NGOR)

**DOI:** 10.3390/cancers17152541

**Published:** 2025-07-31

**Authors:** Mahendra Naidoo, Clare L Scott, Mike Lloyd, Orla McNally, Robert Rome, Sharnel Perera, John R Zalcberg

**Affiliations:** 1 School of Public Health and Preventive Medicine, Faculty of Medicine, Nursing and Health Sciences, Monash University, Melbourne, VIC 3004, Australia; mike.lloyd@monash.edu (M.L.); sharnel.perera@monash.edu (S.P.); john.zalcberg@monash.edu (J.R.Z.); 2 Gynaecology Oncology, Epworth Hospital, Melbourne, VIC 3121, Australia; rrome@bigpond.net.au; 3 Oncology, Peter MacCallum Cancer Centre, Melbourne, VIC 3000, Australia; scottc@wehi.edu.au (C.L.S.); orla.mcnally@thewomens.org.au (O.M.); 4 Clinical Discovery Translation Division, Walter and Eliza Hall Institute of Medical Research, Melbourne, VIC 3050, Australia; 5 Department of Obstetrics and Gynaecology, University of Melbourne, Melbourne, VIC 3010, Australia; 6 Obstetrics and Gynaecology, Royal Women’s Hospital, Melbourne, VIC 3050, Australia; 7 Department of Medical Oncology, Alfred Health, Melbourne, VIC 3004, Australia

**Keywords:** ovarian cancer, BRCA, PARP inhibitors, precision oncology, health services research, clinical quality registry, health disparities, Australia

## Abstract

**Background:** The identification of pathogenic variants in the Breast Cancer Genes 1 and 2 (*BRCA1/2*) is a critical predictive biomarker for poly (ADP-ribose) polymerase inhibitor (PARPi) therapy in epithelial ovarian cancer (EOC). The aim of this study is to define real-world rates and determinants of germline and somatic *BRCA1/2* testing and subsequent PARPi utilisation in Australia using a national clinical quality registry. **Methods:** This multi-centre cohort study analysed data from 1503 women with non-mucinous EOC diagnosed between May 2017 and July 2022, captured by the Australian National Gynae-Oncology Registry (NGOR). We evaluated rates of germline and somatic testing and PARPi use, using multivariate logistic regression to identify associated clinical and demographic factors. **Results**: Overall germline and somatic testing rates were 68% and 32%, respectively. For the high-grade serous ovarian cancer (HGSOC) cohort, rates were higher, at 78% and 39%, respectively. Germline testing was significantly less likely for women aged >80 years (OR 0.49), those in regional areas (OR 0.61), and those receiving single-modality treatment. Somatic testing uptake increased significantly following public reimbursement for PARPi (*p* = 0.004). Among eligible women with a newly diagnosed *BRCA* pathogenic variant and advanced disease (*n* = 110), 52% commenced first-line maintenance PARPi. **Conclusions:** This national study offers valuable insights into Australian ovarian cancer care, highlighting opportunities to enhance testing equity for older women (aged >80) and regional patients. Furthermore, it identifies the translation of a positive test into PARPi therapy as a complex area that warrants further collaborative investigation to optimise patient outcomes.

## Simple Summary

The identification of pathogenic variants in *BRCA1* and *BRCA2* is a critical predictive biomarker for the use of PARP inhibitors in women with epithelial ovarian cancer. However, real-world data on the uptake of *BRCA* testing and subsequent biomarker-driven therapy in Australia are not well characterised. This study leverages data from the Australian National Gynae-Oncology Registry (NGOR) to define national rates of germline and somatic *BRCA* testing and PARP inhibitor utilisation and to identify clinical and demographic determinants associated with these practices. Our findings reveal that while testing rates are encouraging compared to international benchmarks, significant disparities exist for older women (>80) and regional patient populations. Furthermore, a noteworthy evidence–practice gap was observed between the identification of a *BRCA* pathogenic variant and the initiation of PARP inhibitor therapy. These findings highlight where targeted health service interventions are needed to ensure all Australian women have equitable access to precision oncology.

## 1. Introduction

The discovery of pathogenic variants (PVs) in the Breast Cancer Gene 1 (*BRCA1*) and Breast Cancer Gene 2 (*BRCA2*) genes, which account for approximately 10–15% of non-mucinous epithelial ovarian cancers (EOC), has revolutionised the management of this disease [1,2,3]. The presence of a *BRCA* PV is a powerful predictive biomarker for response to platinum-based chemotherapy and most importantly, for a significant and sustained benefit from maintenance therapy with poly (ADP-ribose) polymerase inhibitors (PARPi). Landmark trials such as SOLO-1 have demonstrated that maintenance Olaparib offers a remarkable improvement in progression-free and overall survival for women with newly diagnosed, advanced *BRCA*-mutated EOC, establishing PARPi as a cornerstone of standard-of-care treatment [4,5]. Furthermore, identifying a germline *BRCA* PV has profound implications for cascade family testing and cancer prevention strategies [6]. In recognition of this major international bodies, including American Society of Clinical Oncology (ASCO), European Society for Medical Oncology (ESMO), and National Comprehensive Cancer Network (NCCN), have issued clear guidelines recommending universal germline and/or somatic *BRCA* testing for all women with newly diagnosed non-mucinous EOC [7,8,9]. In Australia, government-funded testing is available for all women with high-grade, non-mucinous EOC.

Despite these clear directives, a gap often persists between evidence-based guidelines and their real-world implementation [10,11,12]. International data reveal highly variable testing rates, with recent studies from the United Kingdom and other European nations highlighting different systemic barriers, from laboratory turnaround times to regional access inequities, which often prevent testing rates from reaching 100% [13,14,15]. Factors including age, socioeconomic status, and access to specialist care, are also frequently cited as determinants of uptake [16,17,18].

Moreover, even when testing identifies an eligible patient, the subsequent uptake of PARPi therapy is not guaranteed. Real-world data on PARPi utilisation outside of clinical trials remain sparse, with some US-based reports showing uptake rates as low as 53% [19].

Clinical Quality Registries (CQRs) are powerful tools for monitoring and benchmarking care against best practices [20]. The Australian National Gynae-Oncology Registry (NGOR) is a national CQR that prospectively captures detailed clinical data, enabling a unique opportunity to investigate these evidence–practice gaps on a national scale [21].

In Australia, a comprehensive, national picture of *BRCA* testing and PARPi treatment patterns has, until now, been largely unknown. Understanding these patterns is crucial for identifying inequities, optimising care pathways, and ensuring that the survival benefits demonstrated in clinical trials are translated to the entire patient population. This study leverages the NGOR platform to achieve two primary objectives. Firstly, to define the real-world rates of germline and somatic *BRCA* testing for women with non-mucinous EOC across Australia, and second, to identify the clinical and demographic determinants of testing and to evaluate the subsequent rates of PARPi utilisation in clinically eligible patients.

## 2. Materials and Methods

### 2.1. Study Design and Population

The NGOR is an ongoing, multi-centre observational cohort study capturing prospective and retrospective data. For this analysis, women aged ≥ 18 years with newly diagnosed, non-mucinous EOC between May 2017 and July 2022 from 21 participating Australian hospitals were included. Patients were followed from diagnosis until death or the data cut-off date of 1 July 2022.

### 2.2. BRCA Testing Pathway in Australia

In Australia, *BRCA* genetic testing is typically initiated by the treating oncology team (‘mainstreaming’), which facilitates rapid access for patients [22]. Germline testing is performed on a blood sample to identify inherited pathogenic variants, and in conjunction somatic testing is performed on tumour tissue obtained from surgery or biopsy to identify variants that have arisen within the cancer itself.

### 2.3. Ethics and Data Collection

The NGOR operates under a national ethics framework (Monash University HREC/17/MonH/198 approved on 8 June 2017) with approvals from all relevant institutional ethics committees. An opt-out consent model is used [21]. Trained data managers at each site enter data from patient medical records into a centralised REDCap database. Routine data quality checks are performed.

### 2.4. Data Definitions

Non-mucinous EOC was defined to include primary ovarian, fallopian tube, and peritoneal cancers. Key variables collected at diagnosis included patient demographics, FIGO 2014 stage, [23] tumour grade and histology, and ECOG performance status [24]. Socioeconomic status (SES) was determined using the Index of Relative Socio-economic Advantage and Disadvantage (IRSAD), categorised into quintiles [25]. Geographic remoteness was classified as ‘Metropolitan’ or ‘Regional’ using the Modified Monash Model [26]. Comorbidity was assessed using the Age Adjusted Charlson Comorbidity Index [27]. The primary outcomes were the rates of germline and somatic *BRCA1/2* testing. The key secondary outcome, ‘PARPi use as clinically indicated,’ was defined as the commencement of first-line maintenance PARPi within eight weeks of completing platinum-based chemotherapy for women with stage III/IV high-grade non-mucinous EOC harbouring a *BRCA* PV. This 8-week cutoff was chosen for methodological rigour to align our real-world analysis with the eligibility criteria of the pivotal SOLO-1 clinical trial [5].

### 2.5. Statistical Analysis

Descriptive statistics were used to summarise the cohort. Categorical variables were compared using the Pearson’s chi-squared test. A key time point analysis was performed comparing testing and treatment rates before and after 1 November 2020, the date on which PARPi received public reimbursement funding in Australia. The Mann–Kendall test was used to analyse trends in testing rates over time. Multivariate logistic regression was performed to identify independent determinants of germline and somatic testing. The age cutoff of >80 years was chosen for analysis as it represents a common demarcation for the ‘oldest old’ in geriatric oncology [28] research and because preliminary data exploration showed a pronounced decline in testing for this group. All analyses were conducted using RStudio (Version 2022.12.0), with *p* < 0.05 considered statistically significant. We used complete case analysis for regression models, therefore patients with missing data on testing status were excluded from these analyses.

## 3. Results

### 3.1. Patient Characteristics

Data from 1503 women with non-mucinous EOC were analysed. The median age at diagnosis was 65 years, with the largest proportion of patients in the 60–69 age group (30.5%). Geographically, approximately two-thirds of patients resided in metropolitan areas (65.7%). In terms of tumour characteristics, the disease was most frequently of ovarian origin (66.6%), overwhelmingly high-grade (Grade 3: 83.9%), and of serous histology (77.0%). The majority of women presented with advanced-stage disease (68.2%), with Stage III being the most common presentation (45.4%).

The socioeconomic distribution of 1430 patients was assessed using IRSAD quintiles. The data showed a skew towards higher socioeconomic status, with the largest group of patients (30.8%) belonging to Quintile 5, the least disadvantaged category. Quintiles 3 and 4 accounted for 20.2% and 19.8% of the cohort, respectively. The two most disadvantaged quintiles, 1 and 2, represented the smallest proportions of patients, with 14.7% and 14.5%, respectively.

The Eastern Cooperative Oncology Group (ECOG) performance status was recorded for 1238 patients, with the data indicating that most had a good performance status at diagnosis. A majority of patients had an ECOG score of 0 (53.0%) or 1 (36.3%), while smaller proportions were recorded for ECOG 2 (7.1%), 3 (3.0%), 4 (0.6%), and 5 (0.1%). In terms of patient comorbidities, the Age-Adjusted Charlson Comorbidity Index was assessed for 1503 patients. The comorbidity burden was varied across the cohort, with the largest group presenting with a severe score (36.9%), followed by those with mild (26.3%), moderate (23.6%), and no comorbidities (13.2%).

Of the 1433 patients whose location was recorded, the majority resided in Victoria (828 patients, 57.8%) and New South Wales (376 patients, 26.2%). Together, these two states comprised approximately 84% of the cohort. Smaller contributions came from Tasmania (123, 8.6%) and Western Australia (67, 4.7%). The lowest representation was from South Australia (29, 2.0%), the Australian Capital Territory (7, 0.5%), and the Northern Territory (3, 0.2%). Of the 1429 patients with recorded regional status, the majority (65.7%, n = 939) resided in metropolitan areas, while 34.3% (n = 490) were from regional areas.

A detailed summary is provided in Table 1, including the addition of national benchmark data from the Australian Institute of Health and Welfare (AIHW) [29].

### 3.2. Rates of Germline and Somatic BRCA Testing

**Figure 1:**
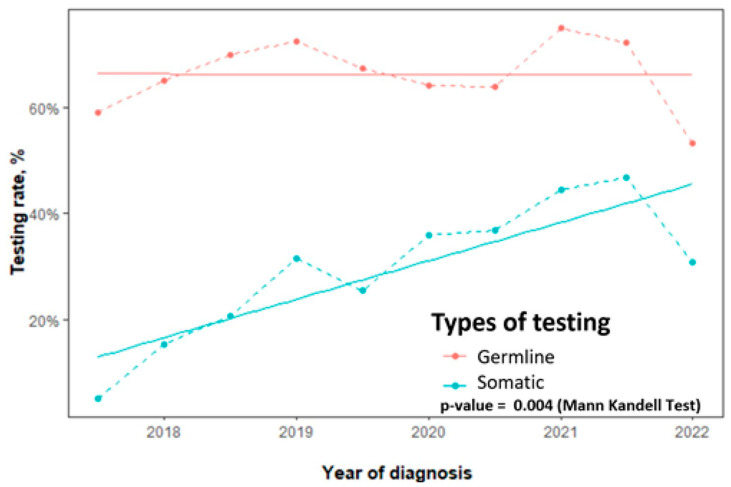
Trends in germline and somatic *BRCA* testing rates for non-mucinous epithelial ovarian cancer, 2017–2022.

Overall, for all non-mucinous EOC, the germline testing rate was 68% (953/1409) and the somatic testing rate was 32% (456/1409). For the key target population of high-grade non-mucinous EOC, the germline testing rate was 72% (849/1179). Among women with high-grade serous carcinoma specifically, germline testing reached 78% (744/954) and somatic testing reached 39% (354/907). The median time from diagnosis to germline testing was 34 days. While germline testing rates remained stable over the study period, somatic testing uptake increased significantly (*p* = 0.004) as shown in Figure 1.

A key time point analysis was conducted to assess the impact of public funding for PARP inhibitors (PARPi), which was introduced on 1 November 2020. Following this date, a statistically significant increase in testing rates was observed across all categories. The rate of germline testing increased from 65.5% (559/853) to 70.9% (394/556), a modest but significant rise (*p* = 0.037). A more pronounced and highly significant increase was seen in somatic testing, which rose from 23.7% (193/814) to 44.2% (234/529) (*p* < 0.001). Consequently, the overall proportion of women receiving either germline or somatic testing also rose significantly, from 67.5% (576/853) before the funding introduction to 77.0% (428/556) after (*p* < 0.001). Detailed results are presented in Table 2.

### 3.3. Determinants of BRCA Testing

**Figure 2:**
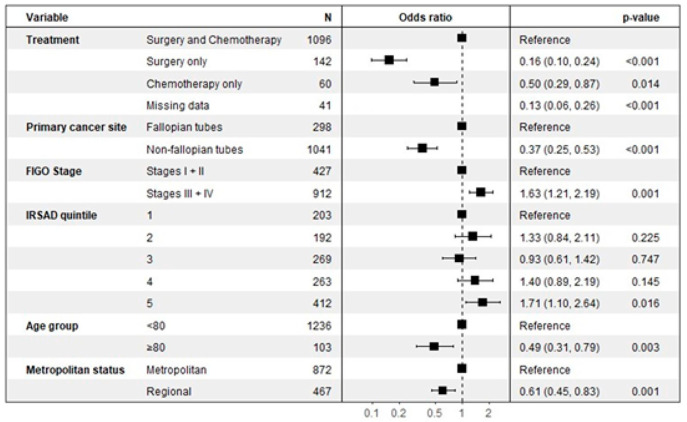
Factors associated with germline BRCA testing.

As detailed in the multivariate analysis shown in Figure 2, several clinical and demographic factors were significantly associated with the likelihood of receiving germline *BRCA* testing.

A patient’s age was a significant determinant, with women aged 80 years and older being approximately half as likely to be tested compared to those under 80 (OR 0.49, 95% CI: 0.31–0.79, *p* = 0.003). Geographic location also played a key role, as women living in regional areas had a significantly lower likelihood of receiving germline testing than those in metropolitan areas (OR 0.61, 95% CI: 0.45–0.82, *p* = 0.001). The planned treatment course was another strong predictor. Compared to patients receiving both surgery and chemotherapy, those managed with single-modality therapy were significantly less likely to be tested, both for patients receiving surgery only (OR 0.16, 95% CI: 0.10–0.24, *p <* 0.001) and chemotherapy only (OR 0.50, 95% CI: 0.29–0.87, *p* = 0.014).

Conversely, women with advanced-stage disease (Stages III or IV) were significantly more likely to undergo germline testing than those with early-stage disease (Stages I or II), with an odds ratio of 1.63 (95% CI: 1.21–2.19, *p* = 0.001). A patient’s socioeconomic status also showed a significant association, where individuals in the highest SES quintile (IRSAD 5) were more likely to be tested compared to those in the lowest quintile (OR 1.71, 95% CI: 1.10–2.64, *p* = 0.016). The primary site of the cancer was also a factor, with non-fallopian tube cancers being less likely to lead to testing compared to cancers originating in the fallopian tubes (OR 0.37, 95% CI: 0.25–0.53, *p* < 0.001).

**Figure 3:**
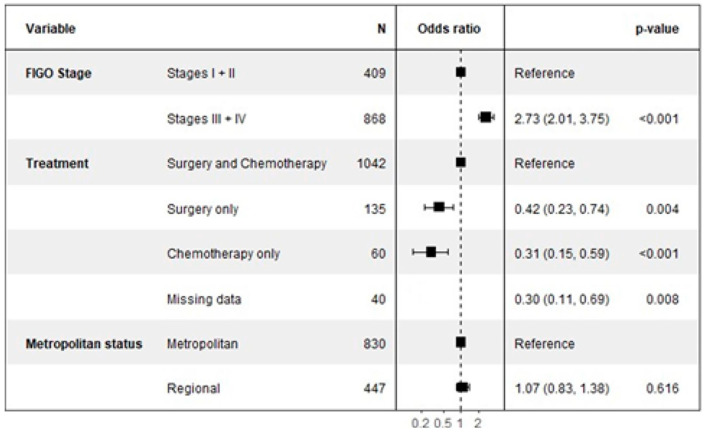
Factors associated with somatic BRCA testing.

The multivariate analysis for somatic *BRCA* testing, detailed in Figure 3, revealed that cancer stage was the most significant predictor of uptake. Patients with advanced disease (Stages III or IV) were nearly three times more likely to undergo somatic testing compared to those with early-stage disease (Stages I or II), with an OR of 2.73 (95% CI: 2.01–3.75, *p* < 0.001).

Conversely, the type of treatment received was strongly associated with a decreased likelihood of somatic testing compared to the reference group who received both surgery and chemotherapy. Patients who received surgery only were significantly less likely to be tested (OR 0.42, 95% CI: 0.23–0.74, *p* = 0.004), as were those who received chemotherapy only (OR 0.31, 95% CI: 0.15–0.59, *p* = 0.001).

Notably, and in contrast to germline testing, a patient’s geographic location was not a significant determinant for somatic testing. There was no statistical difference in the likelihood of receiving a somatic test for patients in regional areas compared to those in metropolitan areas (OR 1.07, 95% CI: 0.81–1.38, *p* = 0.616).

### 3.4. Rates of PARP Inhibitor Utilisation

The rate of first-line maintenance PARP inhibitor (PARPi) utilisation was determined for eligible women with stage III/IV high-grade non-mucinous EOC and an identified *BRCA* pathogenic variant. Overall, 52% (57/110) of this cohort commenced PARPi therapy as clinically indicated. A time point analysis was performed to compare rates before and after public funding was introduced on 1 November 2020. The PARPi uptake rate was 49% in the period before this date and increased to 58% after this date. This observed increase was not statistically significant.

## 4. Discussion

This large, national registry study provides a valuable real-world assessment of *BRCA* testing and PARPi utilisation for ovarian cancer in Australia, highlighting both our collective successes and opportunities for further enhancement of clinical practice. Our findings demonstrate that Australia has achieved encouragingly high rates of genetic testing compared to some international cohorts, while also identifying areas where we can work to close evidence–practice gaps.

Our overall germline testing rate of 68% (78% for HGSOC) is a marked improvement over the 39% reported in the large US-based SEER registry study and approaches the >80% rates achieved in the highly organised Dutch healthcare system [17,30]. This positions Australian practice favourably within the global landscape and reflects a strong commitment to genomic medicine. Interestingly, data from other single-payer healthcare systems like Canada report near-universal testing but highlight different systemic barriers, such as laboratory turnaround times, rather than geographical access, suggesting that local context is critical [31]. A prior single-institution study from Western Australia reported near-universal testing, and our multi-centre national data provide a broader perspective, suggesting that while excellent performance exists, there is natural variation across the country that a national registry is uniquely positioned to explore [32].

The disparity between the high germline testing rate (78%) and lower somatic testing rate (39%) in the HGSOC cohort warrants discussion. This gap may be due to logistical challenges such as obtaining adequate tumour tissue, particularly after neoadjuvant chemotherapy, longer laboratory turnaround times, and a historical clinical focus on germline testing for its familial implications. The NGOR does not collect data on why testing (either germline or somatic) is not performed, and this may be an area for future development.

A key contribution of this study is the identification of opportunities to improve equity in care. The observed lower rates of germline testing among older women (aged > 80), those residing in regional areas, and those managed with single-modality treatment suggest that certain patient groups may face barriers to testing. While clinicians may understandably deprioritise testing in women presumed unfit for aggressive treatment, the profound implications of a germline *BRCA* diagnosis for family members mean that testing remains highly relevant regardless of the patient’s own treatment path. The disparity for regional patients is a well-recognised challenge in a country with a vast geography like Australia and underscores the ongoing importance of initiatives aimed at improving access to centralised specialist and genetic services, a finding echoed in other cancer care studies [33,34].

The finding that women with advanced-stage disease were more likely to undergo germline testing than those with early-stage disease (OR 1.63) reflects the immediate and critical therapeutic implications of a *BRCA* diagnosis in this setting. For women with advanced disease, identifying a pathogenic variant is essential for determining eligibility for first-line maintenance PARP inhibitor therapy, a treatment paradigm proven to dramatically improve survival outcomes [5]. In contrast, for early-stage disease where prognosis is often excellent with surgery alone, the clinical urgency for a *BRCA* result to guide initial treatment is lower. In this context, testing is often viewed through the lens of secondary prevention and familial risk assessment rather than immediate therapy selection. This treatment-focused approach to testing is a common theme in real-world practice, though guidelines from bodies like ASCO recommend testing for all patients with epithelial ovarian cancer, irrespective of stage [7].

This study also identified a significant socioeconomic disparity, where women in the highest SES quintile were more likely to receive germline testing than those in the lowest quintile. This is a critical finding that aligns with international research highlighting the disparities in cancer genetics, where patients may miss out on testing at multiple steps due to socioeconomic factors. Other population-based studies have similarly found that lower socioeconomic status and being uninsured are associated with lower rates of genetic testing for ovarian cancer [16,18,35]. These disparities may be driven by multiple factors, including differences in health literacy, the ability to navigate complex referral pathways, and potential implicit biases within the healthcare system. Addressing these inequities is essential to ensure that all eligible women, regardless of their socioeconomic background, can benefit from the advances of precision oncology.

The study demonstrates an ability to measure the near-immediate impact of health policy on clinical practice through real-world data. The significant increase in somatic testing following public reimbursement for PARPi therapy (from 24% to 44%) provides powerful evidence that funding policy directly shapes clinical practice, showcasing the value of CQRs as tools for health services research and policy evaluation. This finding suggests a pragmatic, treatment-focused approach to testing, where the availability of a funded therapy becomes a key driver for biomarker adoption. As the successful integration of new therapies is a complex process with numerous potential barriers, our data provide a tangible, system-level feedback loop confirming that reimbursement is a powerful, if not essential, lever for the adoption of new oncologic therapies. While this linkage is understandable, it is important to continue promoting testing for its independent prognostic and familial risk implications as well. In contrast, the well-established mainstreaming genetic testing [22], where treating oncologists initiate the process, has likely contributed to the stable, relatively high rates of germline testing from the study’s outset, though our data suggest this has not been sufficient to overcome all barriers for every patient group.

Perhaps the most nuanced finding is that, within this dataset, 52% of women with a newly diagnosed, advanced *BRCA*-mutated EOC commenced first-line maintenance PARPi within 8 weeks of completing chemotherapy. It is important to interpret this figure with caution. The cohort of eligible patients is small (n = 110), and the 8-week cut-off for initiation is stringent and may not capture all patients who eventually started therapy. This registry-based analysis could not account for granular clinical reasoning, and the decision against PARPi may be due to unmeasured factors such as residual toxicity from chemotherapy, patient preference against further treatment, rapid disease progression, or other clinical contraindications not captured in the registry. This figure is similar to the 53% uptake reported in a US real-world study, Ref. [19] suggesting this is a complex international issue. Rather than a simple metric of success or failure, this finding should be viewed as identifying a noteworthy gap between diagnosis and therapy initiation. It presents a valuable opportunity to collaboratively investigate the underlying factors through qualitative studies and targeted quality improvement initiatives.

The primary strength of this study is its large, prospectively collected, multi-centre national dataset, which provides a robust and generalisable snapshot of real-world care. This study has limitations inherent to registry data. This includes the potential for missing data. While we used complete case analysis, this can introduce selection bias, and this should be considered when interpreting the findings. While NGOR and other Clinical Quality Registries in Australia provide robust data, they are not mandated as they are in some European countries, which can further reduce missing data [36,37]. Our cohort included other non-mucinous histologies, but the numbers for individual subtypes like endometrioid were too small for a statistically robust, stratified analysis; therefore, PARPi uptake is primarily generalizable to the high-grade serous carcinoma population. Finally, the study period (2017–2022) overlapped with the COVID-19 pandemic, which may have acted as an unmeasured confounder by impacting healthcare delivery, including potential delays in diagnosis, testing, and treatment.

## 5. Conclusions

This national study provides an important snapshot of genomic medicine in Australian gynaecologic oncology, highlighting established strengths and identifying opportunities to further enhance our equitable and comprehensive delivery of care. A significant proportion of women, particularly older women (aged > 80) and those in regional areas, appear to receive *BRCA* testing at lower rates. Furthermore, the data suggest a complex relationship between a positive test result and the initiation of PARPi therapy. These findings present a national opportunity for collaboration. Targeted educational interventions for clinicians, streamlined referral pathways, and innovative models of care, such as telegenetics, are needed to continue closing the testing gap for underserved populations. Future work should focus on a deeper, more qualitative understanding of the barriers to PARPi uptake. As the landscape evolves to include HRD testing and combination therapies, the role of clinical quality registries like NGOR will be indispensable for monitoring the implementation of these complex new standards and ensuring that every woman with ovarian cancer has access to the best possible personalised care.

## Figures and Tables

**Table 1:** Patient characteristics at time of diagnosis compared with national benchmarks.

**Characteristic**	**NGOR Cohort N (%)**	**AIHW National Benchmark (%) ^1^**
Age (N = 1435)		
<50	172 (12.0%)	15.10%
50–69	753 (52.5%) ^2^	48.30%
70–79	373 (26.0%)	24.50%
>80	137 (9.5%)	12.10%
Primary site of tumour (N = 1498)		NA
Ovary	998 (66.6%)	
Fallopian tube	311 (20.8%)	
Not determined	115 (7.7%)	
Peritoneum	74 (4.9%)	
Tumour Grade (N = 1419)		NA
Grade 1	138 (9.7%)	
Grade 2	52 (3.7%)	
Grade 3	1191 (83.9%)	
Not stated	34 (2.4%)	
Anaplastic	4 (0.3%)	
Cancer Stage (N = 1414)		
I or II	385 (27.2%)	26.00%
III	642 (45.4%)	48.00%
IV	323 (22.8%)	20.00%
Not Stated/Listed	64 (4.5%)	6.00%
Tumour Histology (N = 1404)		NA
Serous carcinoma	1081 (77.0%)	
Endometrioid adenocarcinoma	135 (9.6%)	
Clear cell carcinoma	96 (6.8%)	
Carcinosarcoma	37 (2.6%)	
Mixed cell adenocarcinoma	25 (1.8%)	
Unspecified/Other	30 (2.1%)	

^1^ AIHW (Australian Institute of Health and Welfare) 2024. Cancer data in Australia. Cat. no. CAN 122. Canberra: AIHW. Proportions are for ovarian cancer cases diagnosed between 2018 and 2022. ^2^ The NGOR cohort age groups ‘50–59′ (316; 22.0%) and ‘60–69′ (437; 30.5%) were combined to allow for comparison with the AIHW ‘50–69′ age bracket. NA = not applicable, as directly comparable national benchmark data are not available for these specific cohort characteristics.

**Table 2:** Rates of BRCA testing before and after 1 November 2020 for all non-mucinous EOC.

Characteristic	Before 1 November 2020	After 1 November 2020	*p*-Value ^1^
Germline Testing	(n = 853)	(n = 556)	0.037
Yes	559 (65.5%)	394 (70.9%)	
No	294 (34.5%)	162 (29.1%)	
Somatic Testing	(n = 814)	(n = 529)	<0.001
Yes	193 (23.7%)	234 (44.2%)	
No	621 (76.3%)	295 (55.8%)	
Either germline or somatic testing	(n = 853)	(n = 556)	<0.001
Yes	576 (67.5%)	428 (77.0%)	
No	277 (32.5%)	128 (23.0%)	

^1^ Pearson Chi-squared test.

## Data Availability

Data are unavailable due to privacy and ethical restrictions related to patient confidentiality.
